# A Text Book of Anatomy, Etc.

**Published:** 1854-04

**Authors:** 


*A Text Book of Anatomy*, and guide in dissections for the use of Students of Medicine and Dental Surgery. By Washington R. Handy, M. D., Prof. of Anatomy and Physiology in the Baltimore College of Dental Surgery, etc.

This is an elegant work, containing 785 pages, with 264 woodcut illustrations.

While this branch of Science, in its present state of perfection and completeness, offers but slight opportunity for original contribution, yet we have long entertained the opinion that much might be done, not only in the way of an improved arrangement of the subject, but more especially by rendering its dry and rugged details, in language more attractive; and we believe this object has been aimed at, if not fully attained in the work before us; the style being easy and familiar, while the minutest details are set forth clearly and definitely.

Although this work is professedly designed to meet the wants of both Medical and Dental Students, yet the demand particularly originates with the latter, who have long expressed the want of a production more adapted to their profession than the ordinary works on Anatomy; and in meeting this want the author has given special attention to the anatomy of the mouth, together with its anatomical and physiological relations to the general system.

The author commences with a lengthy introduction, comprising a history of the science of anatomy, and a general outline of organization, while the body of the work is divided into four parts.

The first treats of the elementary tissues; their anatomy, origin, physical and vital properties, etc.

The second part treats of the head; combining in the description of the various organs, both their anatomy and physiology, together with their functional and anatomical relations.

The third part describes the trunk, in the same physiological order; ending by showing the relations existing between the mouth and its several organs and viscera.

The fourth part treats of the extremities in the ordinary manner.

Altogether we believe this work to be, in many respects, a decided improvement, and feel safe in recommending it to the professional public, not only for its accuracy, but more especially the attractive style, in which the author treats his subject, a great desideratum with a large proportion of Students, who are apt to come to the conclusion that "Anatomy is a dry study." F.

